# Supplementary figures and images for: Change of niche in guanaco (Lama guanicoe): the effects of climate change on habitat suitability and lineage conservatism in Chile
Source: PeerJ. 2018 May 28;6:e4907. doi: 10.7717/peerj.4907 (PMC5978400; doi:10.7717/peerj.4907)

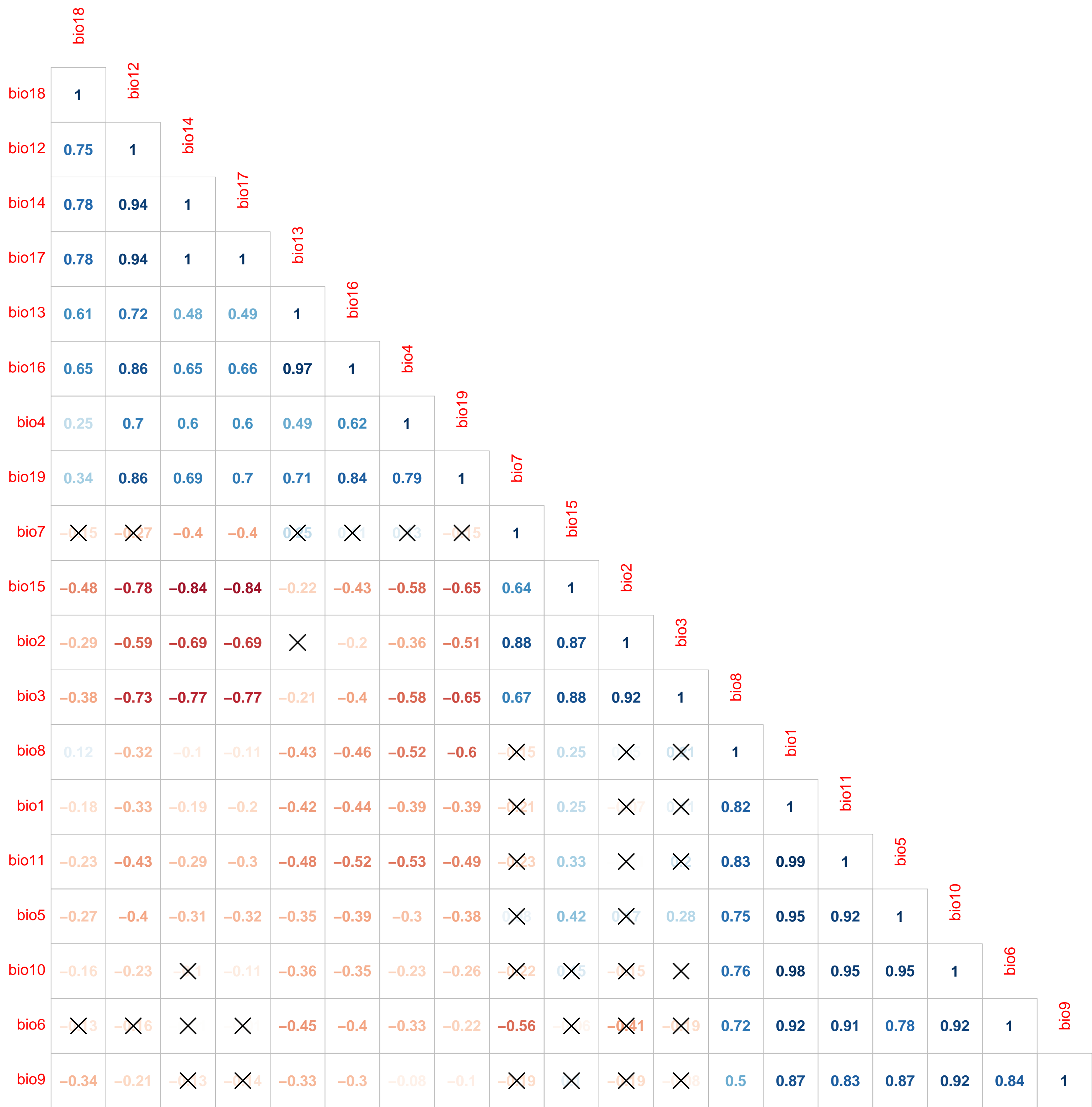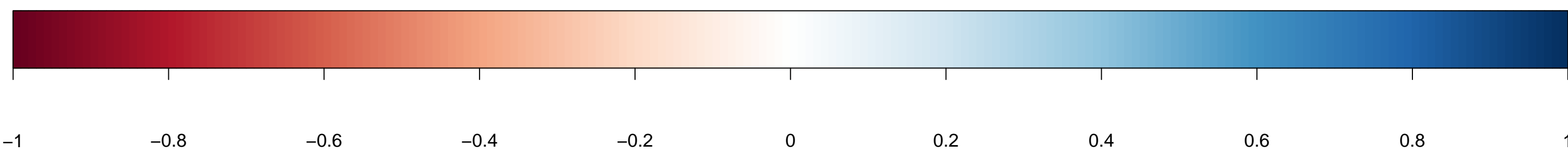

Supplement: Supplemental Information 2 — Pairwise correlation values from bioclimatic variables at guanaco occurrence locations in Chile. Bioclimatic variables are from Worldclim version 1.4. BIO1: Annual mean temperature; BIO2: Mean diurnal range; BIO3: Isothermality; BIO4: Temperature seasonality; BIO5: Max temperature of warmest month; BIO6: Min temperature of coldest month; BIO7: Temperature annual range; BIO8: Mean temperature of wettest quarter; BIO9: Mean temperature of driest quarter; BIO10: Mean temperature of warmest quarter; BIO11: Mean temperature of coldest quarter; BIO12: Annual precipitation; BO13: Precipitation of wettest month; BIO14: Precipitation of driest quarter; BIO15: Precipitation seasonality; BIO16: Precipitation of wettest quarter; BIO17: Precipitation of coldest quarter; BIO18: Precipitation of warmest quarter; BIO19: Precipitation of coldest quarter. [file peerj-06-4907-s002.pdf]

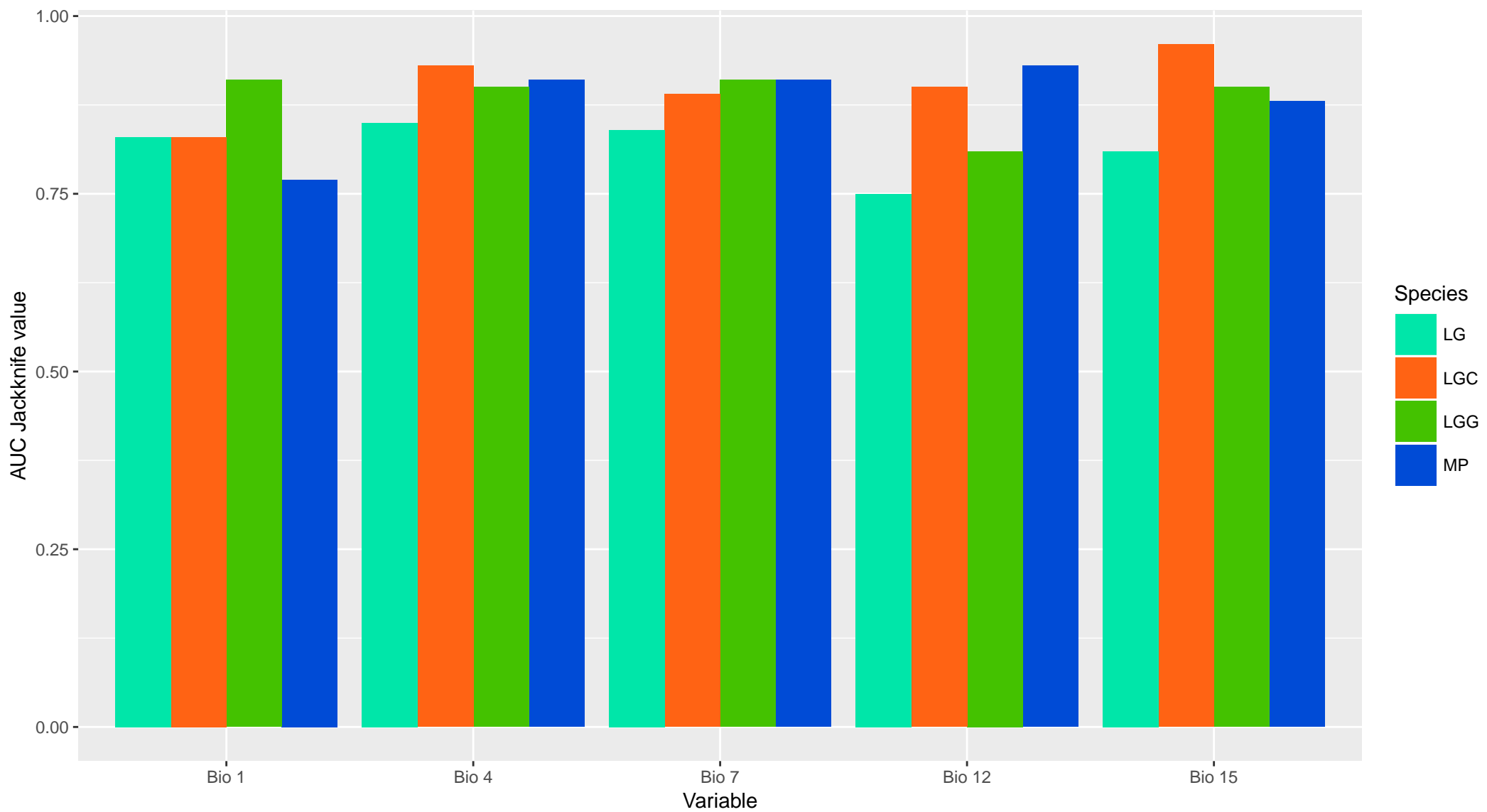

Supplement: Supplemental Information 3 — AUC Jackknife analysis for the environmental variables used in the current distribution models generation of L. guanicoe (LG) and its lineage in Chile (LGC = L.g. cacsilensis; LGG = L.g. guanicoe; MP = Mixed population). The variables are Bio1 = Annual mean temperature; Bio4 = Temperature seasonality; Bio7 = Temperature annual range; Bio12 = Annual precipitation Bio15 = Precipitation seasonality. [file peerj-06-4907-s003.pdf]

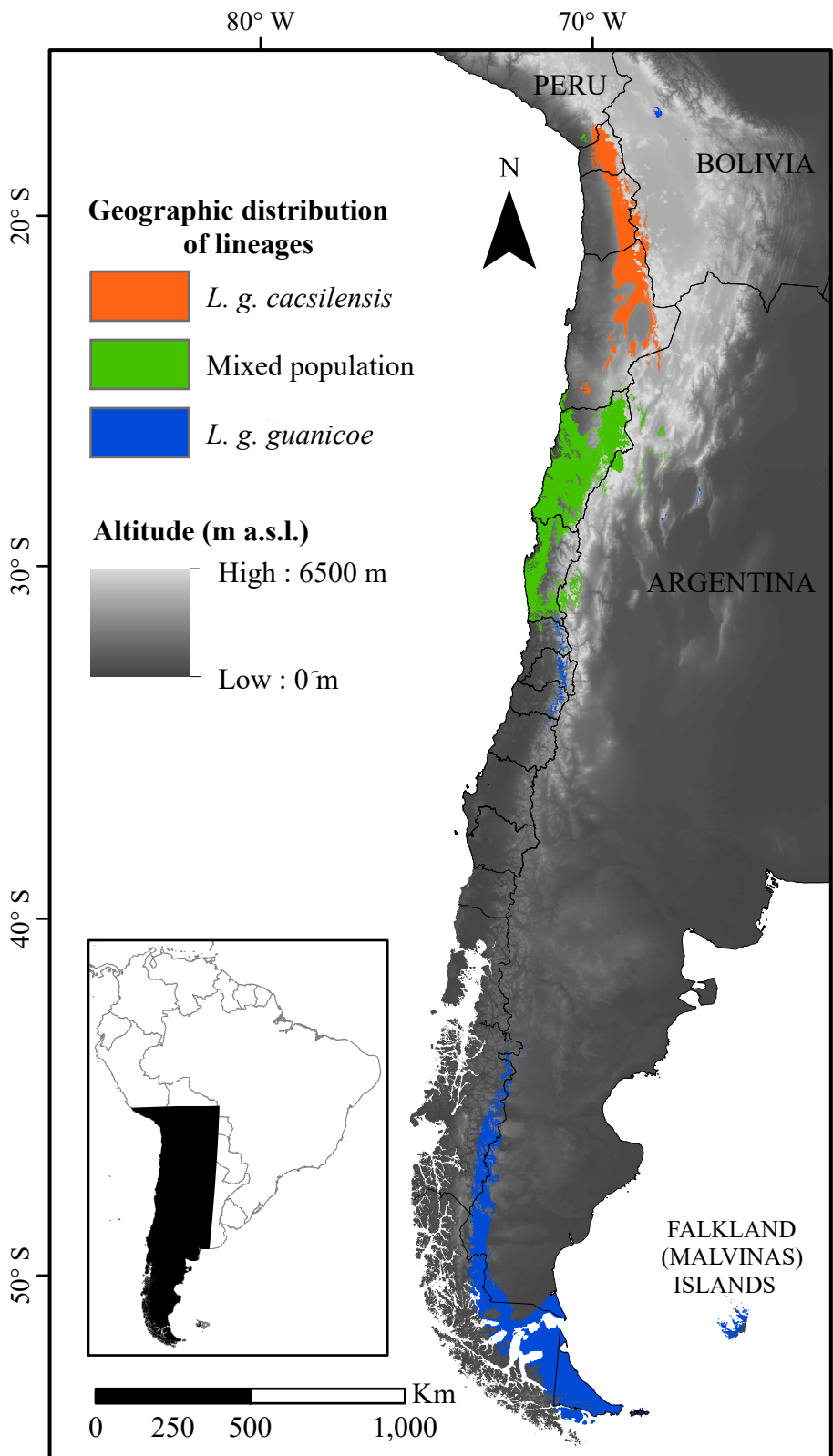

Supplement: Supplemental Information 4 — Base Map Elevation Data: CIAT-CSI SRTM (http://srtm.csi.cgiar.org). [file peerj-06-4907-s004.pdf]
